# Measuring School Absenteeism: Administrative Attendance Data Collected by Schools Differ From Self-Reports in Systematic Ways

**DOI:** 10.3389/fpsyg.2019.02623

**Published:** 2019-12-03

**Authors:** Gil Keppens, Bram Spruyt, Jonas Dockx

**Affiliations:** ^1^Research Group TOR, Department of Sociology, Vrije Universiteit Brussel, Brussels, Belgium; ^2^Centre for Educational Effectiveness and Evaluation, Faculty of Psychology and Educational Sciences, KU Leuven, Leuven, Belgium

**Keywords:** school attendance problems, early identification, truancy, school refusal, school withdrawal, attendance data

## Abstract

In order to use attendance monitoring within an integrative strategy for preventing, assessing and addressing cases of youth with school absenteeism, we need to know whether the attendance data collected by schools cover all students with (emerging) school attendance problems (SAPs). The current article addresses this issue by comparing administrative attendance data collected by schools with self-reported attendance data from the same group of students (age 15–16) in Flanders, the Dutch-speaking part of Belgium (*N* = 4344). We seek to answer the following question: does an estimation of unauthorized absenteeism based on attendance data as collected by schools through electronic registration differ from self-reported unauthorized absenteeism and, if so, are the differences between administrative and self-reported unauthorized absenteeism systematic? Our results revealed a weak association between self-reported unauthorized school absenteeism and registered unauthorized school absenteeism. Boys, students in technical and vocational tracks and students who speak a foreign language at home, with a less-educated mother and who receive a school allowance, received more registered unauthorized absences than they reported themselves. In addition, pupils with school refusal and who were often authorized absent from school received more registered unauthorized absences compared to their self-reported unauthorized school absenteeism. In the discussion, we elaborate on the implications of our findings.

## Introduction

School absenteeism is a serious problem among youth. Youth with *school attendance problems* (SAPs) report lower academic efficacy, poorer academic performances, more anxiety, more symptoms of depression and less self-esteem (Kearney, 2008; Reid, 2014). In addition, school absenteeism is often embedded in a broader pattern of social deviant behavior: youth with attendance problems have an increased risk of stealing, getting involved in vandalism and are more likely to partake in behaviors at the risk of their health (e.g., smoking, substance use; Maynard et al., 2012; Reid, 2014). These specific problems may in turn reinforce long-term SAP and give rise to a vicious circle eventually increasing the risk of early school leaving and later unemployment (Archambault et al., 2009; Rumberger, 2011; Cabus and De Witte, 2015). Hence, early identification of youths with relatively new absentee problems is paramount to prevent more severe and enduring SAPs (Kearney and Graczyk, 2014; Ingul et al., 2019).

In order to optimize identification of youth with (relatively new) absentee problems, many countries invest in attendance monitoring through centralized student management systems. Daily monitoring of students’ attendance is used to ensure fast detection and to enable schools to adopt strategies to intervene when youth have emerging SAPs. More recently, it has been emphasized that in order to maximize early identification of attendance problems, schools need to make better use of their data by also analyzing their collected attendance data (Reid, 2014; Kearney, 2016; Chu et al., 2019). Reid (2014), for example, stresses that an analysis of school attendance data enables schools to identify the causes and school-specific issues of absenteeism. Attendance data can be produced weekly, monthly or yearly and can indicate trends between classes and types of attendance (e.g., seasonal attendance, luxury absenteeism). By using this information, schools can optimize early interventions and create tailor-made strategies. Similarly, Chu et al. (2019) assert that actively analyzing attendance data enables schools to provide attendance feedback to key stakeholders such as students, parents, and counselors. Accordingly, they can use this data to create individualized intervention plans for students or use the data as part of comprehensive school interventions. The extent to which schools maximize the potential of attendance data, however, depends on certain preconditions. This obviously includes the degree of data literacy of the school actors involved (Mandinach, 2012), but also a good understanding of the collected data. Understanding the nature of absenteeism at a school is a crucial first step to appoint more targeted, individualized interventions. To ensure that this process runs efficiently, however, it is important to assess whether certain groups of students are more or less likely to be present in these registration data, compared to information they report themselves. Indeed, in order to apply attendance monitoring within an integrative strategy for preventing, assessing and addressing cases of youth with school absenteeism (cf. Kearney, 2016), we need to know whether the attendance data collected by schools covers all students with (emerging) SAPs.

This article contributes to the aforementioned literature by comparing administrative attendance data collected by schools with self-reported attendance data from the *same* group of students in Flanders, the Dutch-speaking part of Belgium (*N* = 4344). As far as we know, this study is novel in investigating this relationship. The key questions concern whether an estimation of unauthorized absenteeism based on attendance data as collected by schools through electronic registration differs from self-reported unauthorized absenteeism. And if so, whether any differences between administrative and self-reported unauthorized absenteeism are systematic? In other words, are there specific groups of students who are systematically under or overrepresented according to the chosen measurement technique? The latter would indicate that certain types of (emerging) SAPs are more or less prevalent in administrative attendance data when compared to self-reported data.

## Strengths and Limitations of Administrative and Self-Report Attendance Data

School absenteeism is generally measured by means of one out of three different types of data collection strategies: surveys, registration data from school administration or through secondary sources (parents, peers). In this study we focus on self-reported school absenteeism and administrative school attendance data. This section briefly reviews the strengths and limitations of both measurement techniques. Rather than providing a general overview of the strengths and limitations of the data types, we primarily aim to inventory reasons to expect that attendance data as collected by schools (will not) cover *all* students with (emerging) SAPs. This focus on registration data is justified by the fact if schools aim to include data in their school policies, they are most likely to rely on registration data. Furthermore, we want to know which *specific groups* of students are more or less likely to be present according to the measurement technique.

### Administrative Data on School Attendance

Analyses on administrative data of school attendance rely on absences that are recorded by the school staff. In most countries, teachers register school attendance for all students per lesson or per (half) school day. Attendance is monitored by administrative assistants who define whether an absence is (un)authorized and notify school counselors when students exceed a certain threshold of unauthorized absences. Obviously, only those absences that are effectively detected by the school (and defined as unauthorized) are included in administrative data. One strength of administrative data is that they are collected for all students. This implies, for example, that unlike self-reported survey data (see next section), administrative data on school attendance also contains information on groups of students who represent only a very small percentage of the total student population (i.e., students with a specific ethnic background or special needs). Nevertheless, administrative data suffer from at least two limitations.

First, in certain situations, a registered unauthorized absence has little to do with a young person not going to school while having the opportunity to do so. This concerns, for example, absences due to illness which are not justified through a doctor’s note and/or parental consent for the absence. In particular, the latter might apply to children living in low income households due to the financial costs of medical consultation. In such cases, administrative school attendance data are likely to overestimate the level of unauthorized absences from school in a non-random way.

Secondly, there are also indications that official statistics underestimate the amount of absenteeism which is taking place in schools because certain categories of absenteeism remain undetected or are falsely reported as authorized. The first category concerns pre-planned school absenteeism during specific lessons or with specific teachers for which the risk of getting caught is known to be limited. In this context, Reid (1999) distinguishes between specific lessons absenteeism and post-registration truancy. Specific lesson absenteeism refers to the chronic skipping of a specific subject area due to content or the instructor. According to Reid (1999), specific lesson absences originate from a negative student-teacher relationship or dislike of the subject. Keppens and Spruyt (2016, 2017a) argue that it may also be due to an estimated low probability of getting caught whereby some students take advantage of teachers who are sloppier in the registration of absences. Post-registration truancy refers to truancy that occurs *after* students are registered as being present at school (O’Keefe, 1993; Reid, 1999; Keppens and Spruyt, 2016). Hence, post-registration truancy can be considered a specific type of pre-planned specific lesson absence.

A second category of a type of school absenteeism that is more likely to be registered as an authorized absence from school is due to parental consent for the absence. In the first place, this concerns school withdrawal, defined by Heyne et al. (2019, p. 23) as an absence which is (a) not concealed from the parent(s) and (b) attributable to active parental effort to keep the young person at home, or little or no parental effort to get the young person to school. Absenteeism with parents’ knowledge but not consent is called school refusal. The latter refers to a refusal to attend school (a) in conjunction with emotional distress, (b) with parents’ knowledge, (c) without display of antisocial behavior or (d) when parents have made reasonable efforts or express their intention to secure attendance at school (Heyne et al., 2019, pp. 22–23).

### Self-Reported Attendance Data

In the literature, school absenteeism is most often measured through self-reported data (Maynard et al., 2012; Havik et al., 2015; Keppens and Spruyt, 2016), irrespective of whether it is combined with reports from the parents (Kearney and Silverman, 1993; Kearney, 2002). In these studies, young people themselves indicate whether or not they missed school. One of the main strengths of the self-report method is the capacity to investigate the etiology of school absenteeism by means of collecting comprehensive information on individual, familial, school and societal characteristics and influences. The self-report method allows differentiation between different types (e.g., truancy, school refusal, specific lesson absence, school withdrawal), and reasons for (the maintenance of) SAPs (Kearney, 2007; Keppens and Spruyt, 2016; Heyne et al., 2019). This enables one to grasp certain types of school absenteeism (e.g., pre-planned truancy, school refusal) which are difficult to detect in registration data. Hence, one could argue that the measurement of school absenteeism through the self-report method complements administrative school attendance data. However, authors also indicate that self-reported measures of school absenteeism are plagued with a number of problems, resulting in under- or over-reporting.

First, measuring unauthorized school absenteeism through the self-report method may introduce problems because the aim is to gauge behavior that is deviant or delinquent. For example, truancy, defined by Heyne et al. (2019, p. 23) as an absence which occurs (a) when a young person is absent from school for an entire day or part of the day, or at school but absent from the proper location, (b) without the permission of the school authorities and (c) when the young person tries to conceal the absence from their parents, is considered a status offense (Zhang et al., 2007). Hence, respondents are more likely to conceal or fail to recall their truancy out of fear of the consequences, resulting in an underestimation of the actual truancy rate. In this context, research suggests that this underestimation is structurally higher among ethnic minority youth (Kirk, 2006; van Batenburg-Eddes et al., 2012). For example, a Dutch study investigating the discrepancy between self-reported juvenile delinquency and official police statistics found that, in particular, Moroccan youth are less inclined to admit delinquent behavior. The study also showed that this is due to (a) discrimination by the police and (b) a higher level of suspicion toward the authorities due to higher feelings of stigmatization (van Batenburg-Eddes et al., 2012). The same reasoning may apply to the self-reporting of unauthorized absenteeism, and particularly truancy. Zhang (2003), for example, problematizes the subjectivity in authorizing absences since the attendance regulations stipulate that it is up to the school staff to decide which absence should be authorized. In these circumstances, it is plausible that certain students (whose school absenteeism is accompanied by other school misbehavior) or certain types of absences (truancy) are more easily registered as unauthorized than others. Skiba et al. (2011), for example, show that ethnic minorities in the United States are more likely to be referred for truancy as compared to their white peers (African American youths in grade 6 to 9 are 4.40 times more likely to be referred for truancy than their white peers; Hispanic/Latino youth in grade 6 to grade 9 are 2.44 times more likely to be referred for truancy than their white peers). Skiba et al. (2011) also demonstrated that ethnic minorities are more likely than their white peers to receive expulsion or out of school suspension as a consequence of referred truancy. Hence, ethnic minorities might (compared to their peers without a migration background) be overrepresented in administrative data on absenteeism because of discrimination by the school staff. However, at the same time, ethnic minorities might also be underrepresented in the self-reported school absenteeism data due to feelings of suspicion toward the school authorities when filling in self-reported questionnaires on deviant behavior.

A second limitation of the self-report technique is that it relies on students’ recollections of their absenteeism and this might undermine the reliability of the data. This applies in particular to self-report measures that rely on longer time frames. The longer this period, the greater the chance that the self-reported absenteeism will deviate from the real absenteeism rate (Stone et al., 2000; Kirk, 2006). However, it should also be noted that self-reported measures that use a shorter reference period to measure absenteeism (for example, 2 weeks) may lead to an underestimation of school absenteeism. When the reference period is short, there will likely be an underreporting of students who are only absent a few times a year (Keppens and Spruyt, 2017b).

### The Current Study

The preceding arguments suggest that self-reported data and administrative data on school absenteeism are each associated with some advantages and disadvantages due to their specificity. The added value of self-reported data on school absenteeism is that it enables stakeholders to assess absenteeism in more detail. Certain types of absences that remain invisible in administrative data on absenteeism are more likely to be grasped with the self-report technique. In this way, self-reported data on school absenteeism provide an indication of the extent to which administrative data on absenteeism cover all students with (emerging) SAPs. Against this background, this paper is the first study that compares self-reported data on school absenteeism with administrative data of unauthorized absences among (the same group of) students from the fourth year of secondary education in Flanders. More specifically, we investigate: (1) the extent to which self-reported data on school absenteeism and administrative data of unauthorized absences gauge the same behavior, and (2) the extent to which possible discrepancies are related to the type of school absenteeism (e.g., truancy, school refusal, school withdrawal, pre-planned truancy and authorized school absenteeism) and students’ characteristics (in particular, ethnicity and SES).

## Materials and Methods

### Study Design

To answer our research questions, we merged self-reported data on school absenteeism from the longitudinal LiSO (Educational Trajectories in Secondary Education) project with data from the administrative database on absences from the Flemish Ministry of Education and Training (named DISCIMUS in the remainder of this paper).

The LiSO project follows a cohort of 6457 students in 57 schools who started secondary education in the school year 2013–2014 (Stevens et al., 2015). A regional sampling strategy was used whereby nearly all students in the targeted cohort who attended school in the target geographic region were included in the study (Dockx et al., 2019). For the present study, data were used from wave 4 (T4) which was gathered at the end of the fourth year (May 2017) of secondary education (age 15–16). T4 is the only wave that included items gauging self-reported school absenteeism. The total sample of students in T4 consisted of 6545 students in 53 schools. Within this sample, 4344 students completed the questionnaire in a valid way resulting in a total response rate of 66.69%.

Registration data on absences among all students in primary and secondary education are collected by the Flemish Agency for Educational services (AGODI). In Flanders, school attendance is registered twice a day. There are many reasons why a student is absent from school. Absences due to illness (and authorized by a doctor or through a parental note)^1^, a funeral of a relative or religious holidays are authorized. When a student has no justified reason for his/her absence (i.e., has an unauthorized absence from school), s/he receives, per half school day, a so-called “B-code”. Schools automatically exchange these registered absences (all absences including unauthorized absences) within a centralized database (DISCIMUS). This enables the Flemish Ministry of Education and Training to link the collected data to other student characteristics. At any time, schools can request the absences they have registered. As a result, the registration data on school absenteeism in Flanders is not only used to intervene at the level of the students^2^, but also to gain insight into the distribution of all absences across different classes and school years. In general, Flanders can be considered as one of the forerunners in Europe when it comes to the accurate and systematic collection of data on school absenteeism among students who follow compulsory education (European Commission, 2013).

In DISCIMUS, each student has a unique identification number. In this paper, we used this unique identification number to merge data from the DISCIMUS database with data from the LiSO database. Only registrations of unauthorized absences that occurred before filling in the LiSO questionnaire were considered.

Because this study involved students in Flemish secondary education and was an initiative of the Flemish government, approval was required of the Belgian *Commissie voor de bescherming van de persoonlijke levenssfeer* (Commission for the protection of the personal privacy). The Commission approved the data collection of the LiSO-project. Parents and students have been informed yearly, with a personal letter and the *schoolreglement* (school charter). A *schoolreglement* in Flanders is a document that contains the specific regulations of the school and its pedagogical project. It needs to be signed by the parents and the student to declare that they agree with the regulations and pedagogical project of the school. By signing this document, they also agree to participate with the LiSO-project and other studies that the school had chosen to participate in.

However, even after signing to agree with the school charter, parents and students can still choose to opt out of a study. This procedure was also approved by the *Commissie voor de bescherming van de persoonlijke levenssfeer.* The linking of the data of the LiSO-project and DISCIMUS poses no specific issues, for the *Commissie voor de bescherming van de persoonlijke levenssfeer* approved that the data can be linked to other datasets. Furthermore, parents and students were informed in the personal letter and the school charter that such linking of data would occur.

### Questionnaire Data

Self-reported unauthorized school absenteeism was measured through the following question: “How many times did you skip school without a valid reason in the current school year?” Students who reported to have skipped school at least once were asked about whether their parents knew about the absence and if so whether they approved the absence. These characteristics allowed us to differentiate between three types of SAP: truancy, school refusal and school withdrawal (Heyne et al., 2019). In this study, and following Heyne et al. (2019), unauthorized absences that are concealed from the parents were labeled as *truancy*. Unauthorized absences that occurred with knowledge of parents, but without consent were labeled as *school refusal*. Unauthorized absences that occurred with approval of the parents were labeled as *school withdrawal*. In addition, information was gathered on pre-planned truancy and self-reported authorized absenteeism. Pre-planned truancy was measured by asking students who reported to have skipped at least once whether their unauthorized absences were discovered by the school staff. Self-reported authorized absenteeism was measured by asking: “How often were you absent from school for a valid reason this school year due to family or personal reasons (e.g., death of a friend or family member) or illness (I had a valid note from my parents or the doctor)”. Respondents answered on a Likert-scale ranging from 1 (never) to 5 (more than 10 times).

### Administrative Data

Registered unauthorized absences are measured through the number of “B-codes” in the DISCIMUS dataset. A student receives a B-code for each half school day of unauthorized absence. In other words, a student who had an unauthorized absence for a whole school day receives 2 B-codes. The school year 2016–2017 in fulltime secondary education counted 316 half school days, which equals the maximum number of B-codes a student can receive for that school year. The rate of B-codes among the students in our sample ranged from 0 to 101 (*M* = 2.41, *SD* = 6.75). To compare the registered and self-reported unauthorized absences, the following procedure was used. First, every day on which a student was absent for the whole school day (i.e., for which s/he received 2 B-codes) was recoded to 1. Since the self-reported measure of unauthorized absenteeism asks respondents to report *how many times* they skipped school, students who were absent for a whole school day will likely report this as one time. Next, we recoded the number of B-codes to match the categories used in the self-report measure: none, once, 2 times, 3 times, 4 times, 5 times, 6 times, 7 times, 8 times, 9 times, 10 to 15 times, 15 to 20 times, or more than 20 times. In addition, information on the characteristics of the students were obtained, including gender, ethnicity (speaks foreign language at home), age, educational track (general/arts or technical/vocational) and SES. The latter is measured through the educational level of the mother and whether the student receives an education allowance.

### Statistical Analyses

In this study we conducted Poisson multilevel regression analyses (with STATA 14) with the prevalence of registered unauthorized school absences as dependent variable to assess the relationship between self-reported and registered unauthorized school absenteeism. A Poisson model is the most suitable technique since our measures of unauthorized school absenteeism are count variables that are bounded by zero (one cannot be absent from school less than 0 times) and not normally distributed (Cameron and Trivedi, 2013). The multilevel structure enabled us to control for differences between schools (e.g., whether schools are more or less strict in their registration and detection of unauthorized absences). The first model included the sociodemographic variables gender, ethnicity, age, educational level and SES that are known to relate to school absenteeism (Kearney, 2008; Reid, 2014). In the second model we added the prevalence of self-reported unauthorized school absenteeism. This allowed us to assess whether the administrative data under or overestimated the degree of unauthorized school absenteeism of particular social groups, compared to the self-report data. The latter would be the case when some of the sociodemographic variables remained significant *after* taking into account the self-reported absences. Model 2a examines these associations for our total sample (*N* = 4344). Model 2b examines these associations only for those students who reported to have an unauthorized absence from school at least once (*N* = 777). This subsample included students who had valid answers on the self-reported question on unauthorized school absenteeism and all subsequent measures concerning the type of SAPs. In the third model, we analyzed whether the administrative data under or overestimated (when compared to the self-report data) the degree of unauthorized school absenteeism of certain types of school absenteeism by adding the typology of SAPs, pre-planned truancy and authorized school absenteeism.

### Non-response

For the non-response analysis, students who did not (adequately) complete the questionnaire were compared with students who did. Students who did not complete the questionnaire could not because they were absent when their classmates filled in the questionnaires. Some schools were also less motivated to give students sufficient time to properly fill out the questionnaire. Students who failed to complete the questionnaire had statistically more unauthorized absences from school than students who completed a questionnaire, respectively, 13.51 to 2.62 [*F*(1) = 737.58, *p* < 0.001].

## Results

Tables 1, 2 present the characteristics of the study population based upon, respectively, the questionnaire data and the administrative data: 50.4% of the participants were boys, 10.5% spoke a foreign language at home, 18.1% had a less educated mother (not finished secondary education), 23.4% received a school allowance and 50.5% was enrolled in technical or vocational education. The prevalence of registered unauthorized school absenteeism was higher (39.1%) than the prevalence of self-reported school absenteeism (19.2%). Among the group of students who reported to have at least once been unauthorized absent from school, 49.4% could be categorized as truancy, 17.4% as school refusal and 33.2% as school withdrawal. Additionally, 57.8% of the students reported that their unauthorized school absenteeism was never discovered.

**TABLE 1:** Sample characteristics based upon questionnaire data.

	**Percent**	***N***
**Self-reported unauthorized school absenteeism**		4344
Never	80.8	
1 time	9.0	
2 times	2.9	
3 times	2.3	
4 times	1.6	
5 times	0.8	
6 times	0.7	
7 times	0.2	
8 times	0.3	
9 times	0.2	
10 to 15 times	0.6	
15 to 20 times	0.3	
>20 times	0.3	
**Type of school attendence problem (SAP)**		777
Truancy	49.4	
School refusal	17.4	
School withdrawal	33.2	
**Has it ever been discovered that you skipped school?**		777
Never	57.8	
Once	28.8	
Several times	8.5	
Often	2.2	
Always	2.7	
**Self-reported authorized school absenteeism due to family or personal reasons (e.g., death of a family member or a friend)**		4344
Never	3.4	
Once	21.0	
2 to 5 times	42.9	
5 to 10 times	19.3	
>10 times	13.4	

**TABLE 2:** Sample characteristics based upon administrative data.

	**Percent**	***N***
**Registered unauthorized school absenteeism**		4344
Never	61.9	
1 time	14.8	
2 times	7.6	
3 times	3.9	
4 times	2.4	
5 times	1.9	
6 times	1.3	
7 times	1.3	
8 times	0.8	
9 times	0.6	
10 to 15 times	1.7	
15 to 20 times	0.9	
>20 times	0.9	
Gender, boy	50.4	4344
Age		4344
14	0.5	
15	41.5	
16	45.7	
17	10.4	
≥18	1.9	
Ethnicity, foreign language at home	10.5	4344
Educational level of mother, did not obtain diploma secondary education	18.1	4344
School allowance, receives school allowance	23.4	4344
Educational track, technical+vocational	50.5	4344

Table 3 shows the correlation between self-reported and registered unauthorized school absenteeism and helps to answer our first research question. We observed a weak but significant positive correlation (*r_*s*_* = 0.23, *p* < 0.001). The strength of this correlation increased when it was re-estimated among the subsample of students who reported to have an unauthorized absence from school at least once (*r_*s*_* = 0.40, *p* < 0.001). The same observation applies for the group of students who reported to have at least one unauthorized absence from school *and* who have been registered with at least 1 B-code (*r_*s*_* = 0.44, *p* < 0.001). This indicates that the rather weak association between self-reported and registered unauthorized school absenteeism is mainly due to students who have been registered with at least one B-code but do not report to have skipped school. When we omitted this group of students, we found a medium-strong association between self-reported and registered unauthorized school absenteeism.

**TABLE 3:** Spearman correlation coefficients between self-reported and registered unauthorized school absenteeism.

All students	0.23^∗∗∗^
Students who reported to have been at least once unauthorized absent from school	0.40^∗∗∗^
Students with at least 1 B-code	0.23^∗∗∗^
Students who reported to have been at least once unauthorized absent from school and with at least 1 B-code	0.44^∗∗∗^

*^∗∗∗^*p* ≤ 0.001.*

Multivariate analyses enabled us to answer our second research question: whether the observed discrepancies between registration and self-reported data are related to the type of school absenteeism or the student’s characteristics (Table 4). Model 1 confirms earlier research showing that unauthorized school absenteeism is more prevalent among boys, students in technical and vocational tracks and students who speak a foreign language at home and with a low SES (Kearney, 2008; Reid, 2014). Model 2 shows significant associations between all of our inserted student characteristics and registered unauthorized school absenteeism after controlling for self-reported unauthorized school absenteeism. In other words, boys, students in the technical and vocational tracks and students who speak a foreign language at home, with a low-educated mother and who received a school allowance received more B-codes than they reported themselves. The same applied for older students. For model 2b, only students who reported to have an unauthorized absence from school at least once were selected (*N* = 777). We observed no large discrepancies between model 2a and 2b, except for gender^3^. Model 3 indicates that, in particular, students with school refusal received more B-codes compared to their self-reported rate of unauthorized school absenteeism. The same applied for authorized school absenteeism. Students who (often) had authorized absences from school received more B-codes compared to their self-reported unauthorized school absenteeism. Finally, we found that students who pre-planned their school absenteeism and reported that their absenteeism had never been discovered received less B-codes when compared to the rate of unauthorized school absenteeism that they reported themselves.

**TABLE 4:** Results of Poisson multilevel analyses on the association between registered unauthorized school absenteeism, self-reported unauthorized school absenteeism, student’s characteristics and the type of school absenteeism.

	**Model 1**		**Model 2a**		**Model 2b**		**Model 3**	
				
	**B**	**SE**	**B**	**SE**	**B**	**SE**	**B**	**SE**
Intercept	−5.00^∗∗∗^	(0.27)	−4.39^∗∗∗^	(0.27)	–4.38^∗∗∗^	(0.51)	–4.14^∗∗∗^	(0.53)
Gender (0: girl)	0.14^∗∗∗^	(0.03)	0.08^∗^	(0.03)	–0.17^∗∗^	(0.05)	–0.17^∗∗^	(0.06)
Ethnicity (0: speaks no foreign language at home)	0.28^∗∗∗^	(0.04)	0.35^∗∗∗^	(0.03)	0.11(^*^)	(0.07)	0.15^∗^	(0.07)
Educational level of the mother (0: no secondary education)	0.21^∗∗∗^	(0.03)	0.14^∗∗∗^	(0.03)	0.27^∗∗∗^	(0.06)	0.09	(0.06)
School allowance (0: receives no school allowance)	0.28^∗∗∗^	(0.03)	0.30^∗∗∗^	(0.03)	0.13(^*^)	(0.05)	0.26^∗∗∗^	(0.05)
Educational track (0: general/art)	0.73^∗∗∗^	(0.05)	0.65^∗∗∗^	(0.05)	0.61^∗∗∗^	(0.09)	0.54^∗∗∗^	(0.09)
Age	0.27^∗∗∗^	(0.02)	0.23^∗∗∗^	(0.02)	0.26^∗∗∗^	(0.03)	0.25^∗∗∗^	(0.03)
Self-reported unauthorized school absenteeism			0.16^∗∗∗^	(0.01)	0.15^∗∗∗^	(0.01)	0.12^∗∗∗^	(0.01)
SAP type (0: truancy)								
School refusal							0.22^∗∗∗^	(0.07)
School withdrawal							0.01	(0.06)
Discovered unauthorized school absences							–0.06^∗∗^	(0.02)
Authorized school absenteeism							0.05^∗∗∗^	(0.01)
N students	4344		4344		777		777	
N schools	54		54		54		54	
Model deviance	15031.94^∗∗∗^		14025.63^∗∗∗^		3337.98^∗∗∗^		3285.37^∗∗∗^	

*The estimated Poisson regression coefficients (B) are presented with standard errors (SE) and Model Deviance, with significance level of the Chi–squared test comparing it to the deviance of the previous model (except model 2b); Model 1 is compared to the null-model. (^∗^)*p* ≤ 0.10; ^∗^*p* ≤ 0.05; ^∗∗^*p* ≤ 0.01; ^∗∗∗^*p* ≤ 0.001.*

## Discussion

Early identification and intervention of SAPs is crucial to restoring regular school attendance and limiting the long-term impact of these SAPs on students’ educational trajectories. In the literature, much attention has been devoted to so-called Response to Intervention frameworks (RtI), sometimes also referred to as Multi-tiered Systems of Support frameworks (MTTS) (Kearney and Graczyk, 2014; Kearney, 2016; Chu et al., 2019; Heyne, 2019; Ingul et al., 2019). RtI refers to a systematic and hierarchical decision-making process to assign evidence-based strategies based on students’ needs and in accordance with regular progress monitoring. A RtI framework applied to school attendance promotes regular attendance for all students at TIER 1, targeted interventions for at-risk students at TIER 2, and intense and individualized interventions for students with regular absenteeism at TIER 3 (Kearney and Graczyk, 2014; Kearney, 2016). In order to work successfully, the RtI framework relies strongly on a valid and reliable identification and detection system. Only when a new absentee problem is identified, early intervention can be initiated in order to prevent absenteeism becoming more severe and chronic. In the present study, we built on this perspective by assessing the systematic (mis)match between absenteeism as registered by schools compared to self-reports. Based on unique survey data among 4344 students (aged 15–16) that could be linked to administrative data we found a weak correlation between measures of unexcused school absenteeism. Moreover, the mismatch between registration and self-report data was systematic with boys, students in technical and vocational tracks and students who speak a foreign language at home, with a less-educated mother and who receive a school allowance having consistently higher rates of registered unauthorized absenteeism compared to what they reported themselves. In addition, pupils with school refusal and who were often authorized absent from school received more registered unauthorized absences compared to their self-reported unauthorized school absenteeism. What implications do these two key findings have?

First, regarding the weak association between self-reported unauthorized school absenteeism and registered unauthorized school absenteeism, the rate of registered unauthorized school absenteeism was approximately twice as large compared to the rate of self-reported school absenteeism. Several mechanisms may help to explain this discrepancy. Some students pre-plan their truancy and do everything to avoid being caught (Keppens and Spruyt, 2017a). Other students might be more suspicious when they report their unauthorized absences and consequently provide fewer valid responses in a questionnaire. In other cases, the observed discrepancy may be due to biased school staff when deciding whether or not an absence is authorized or due to parents who legitimize the (unauthorized) absences of their children. At the same time, our findings also suggest that in order to optimize the validity and reliability of school attendance identification systems, schools need to actively analyze their attendance data. Indeed, this paper shows that to maximize the potential of attendance data and to ensure that students do not fall between the cracks of the registration system, the mere collecting and monitoring of attendance data is insufficient. Schools also need to analyze their collected data. Only by analyzing the data, trends between types of students and types of attendances can be identified. It is therefore surprising to find that the question “*how to use attendance data at a school level, within a multitier framework*” remains a largely unanswered question in the extant literature. Given the large number of youth with absences [11% of adolescents in the United States between the ages of 12–17 reported skipping school in the past 30 days and 17.82% of the 15-year-old students in the EU reported skipped school in the past 2 weeks (Maynard et al., 2017; Keppens and Spruyt, 2018)], the use of technology to enhance early identification is indispensable. Failing to answer the question how attendance data can be used at schools within a multitier framework may lead to an accountability culture in which the registration of absenteeism becomes and end in itself rather than a starting point to critically reflect on and gain more insight in to the meaning of (emerging) SAPs. This may lead to a situation in which schools are urged to implement registration systems, but lack the sufficient resources and support to guide students with SAPs in a customized way.

Second, in the context of discussions concerning interventions to reduce school absenteeism many authors lament about the lack of a unified approach to differentiate between youth with SAPs (Heyne et al., 2019; Tonge and Silverman, 2019). According to Heyne et al. (2019), differentiation is beneficial because SAPs are heterogenous, varying in etiology and presentation, while having associations with a broad array of risk factors. The authors argue that risk and protective factors associated with the development, maintenance, and prevention of SAPs are likely to be different for different types of SAPs. The most effective interventions might indeed be those that target the factors relevant to a particular type of SAP (see also Heyne, 2019). In order to integrate these perspectives within the RtI framework, we must examine whether certain specific interventions are more effective according to the type of SAP (Tonge and Silverman, 2019). Following the same reasoning, we must also ensure that all types of SAPs are identified in a timely manner through attendance tracking. Concerning the latter, our results suggest that there is a particular discrepancy between self-reported unauthorized school absenteeism and registered unauthorized school absenteeism among students with school refusal. Students with school refusal received more B-codes compared to the rate of unauthorized school absenteeism that they reported themselves. A plausible explanation for this observation is that these students do not perceive their absences as unauthorized and consequently do not report them as such in self-reported questionnaires. In this paper, we measured unauthorized absenteeism by means of an item asking youth whether they have skipped school without a valid reason. As Heyne et al. (2019, p. 7) already pointed out, the notion of skipping school without a valid reason is open to broad interpretation. Students with school refusal could have interpreted their general fear of school as a valid reason to skip school. Interestingly, we did not observe a different association between self-reported absenteeism and registered school absenteeism among students with truancy and students with school withdrawal. For both types of school absenteeism, we expected to find higher rates of self-reported absenteeism compared to the rate of registered school absenteeism. Among students who truant, the association between self-reported and registered school absenteeism is likely interrupted due to pre-planned and premeditated truancy. For those students who withdraw from school, it is probable that the association between self-reported and registered school absenteeism is interrupted by parents legitimizing their children’s absences.

Finally, we acknowledge the limitations of this study. First, as mentioned earlier, this study examines the relationship between self-reported and registered unauthorized school absenteeism while knowing in advance that both are not completely the same. A student who is ill but does not have a doctor’s note will not report that absence as unauthorized, yet it will be registered by the school staff as such. Within the same line of reasoning, some students might perceive reasons for absences as “legitimate” while these are not defined as such by the school. That is why we did not use statistical indicators which measure the degree of agreement (e.g., Kappa’s coefficient) which are often used in criminological research to compare police statistics with self-reported delinquency. In this paper, we primarily focused on the association between self-reported and registered absences and, in particular, on whether some subgroups of students or types of absence are more prevalent in some types of data. The advantage of that strategy (by means of Poisson regression analysis) is that modifications and recoding of the rate of registered absences (see section “Administrative Data”) had no effect on our conclusions. After all, we only divided the rate of unregistered absences through a constant factor. Second, relying on whether parents knew and/or approved of the absence to measure the type of absenteeism may not be optimal. Generally, truancy is characterized by a lack of parental knowledge of the absence, school refusal by parental knowledge without consent, and school withdrawal by a lack of parental consent. However, Heyne et al. (2019) note that in some cases, students with school refusal conceal their non-attendance from their parents (see also: Elliott, 1999). In other cases, parents might be more ambivalent toward their child with school refusal due to “overprotectiveness” of parents who are afraid of pressuring their child too much (Heyne et al., 2019, p. 26). Ideally, questions about a student’s reluctance or refusal to attend school are needed to more accurately differentiate between truancy, school refusal and school withdrawal. Unfortunately, these questions were not included in the self-reported questionnaire. However, these limitations do not alter the fact that this paper is among the first to gauge the prevalence of different types of absences on a large representative sample (*N* = 4344). While the latter was not the objective of this paper, this research suggests, in agreement with research from Berg (2002) and Egger et al. (2003), that the rate of school refusal is less common than truancy. In addition, the results also suggest that the rate of school withdrawal is more prevalent, compared to school refusal and slightly less than truancy. Future research on the prevalence of these types of school absenteeism is needed to strengthen the claims in this paper.

## Conclusion

This study’s main finding is the weak association between self-reported unauthorized school absenteeism and registered unauthorized school absenteeism. The rate of registered unauthorized school absenteeism was approximately twice as large compared to the rate of self-reported school absenteeism. Boys, students in the technical and vocational tracks and students who spoke a foreign language at home, with a low-educated mother and who received a school allowance received more B-codes than they reported themselves. The same applied for school refusal and authorized school absenteeism. Students who pre-planned their truancy, on the other hand, received less B-codes than they reported themselves. More understanding of these discrepancies through future research is needed because it suggests that (1) researchers should be cautious with generalizing scientific research about school absenteeism between self-reported and administered data and (2) school staff and other stakeholders might not reach all students with SAPs when interventions and counseling are exclusively based on the registration of unauthorized absences.

## Data Availability Statement

The datasets generated for this study are available on request to the corresponding author.

## Ethics Statement

Ethical review and approval was not required for the study on human participants in accordance with the local legislation and institutional requirements. Written informed consent to participate in this study was provided by the participants’ legal guardian/next of kin.

## Author Contributions

GK and BS designed and planned the study. GK and JD structured and analyzed the data. GK wrote the manuscript. All authors interpreted the data, took responsibility for the integrity and accuracy of the data analysis and the decision to submit this manuscript for publication, read, and approved the final manuscript.

## Conflict of Interest

The authors declare that the research was conducted in the absence of any commercial or financial relationships that could be construed as a potential conflict of interest.

## Footnotes

1 For an absence up to three consecutive calendar days, a note from the parents is sufficient. A medical certificate from the doctor is required in the following cases: (1) if the student is ill for four or more consecutive calendar days (e.g., Friday, Saturday, Sunday and Monday = medical certificate; (2) for every absence due to illness, no matter how short, if the student was already absent four times in the same school year legitimized through a parental note; and (3) if the student is ill during exam periods.

2 In Flanders, schools screen the nature of each half school day of unauthorized absence from school. When this absence is regarded as high-risk or when students receive at least 5 B-codes, school counselors start up a more individualized approach.

3 Among the subsample of students who reported to have an unauthorized absence from school at least once, in particular, girls seem to have more B-codes compared to what they reported themselves. Subsequent analyses showed an interaction-effect between gender and self-reported unauthorized school absenteeism (results available on request). When the rate of self-reported unauthorized school absenteeism increases, the relationship between self-reported and registered unauthorized school absenteeism is stronger for girls than for boys. A possible explanation is that among students with more severe SAPs, boys are less likely to admit their “deviant” behavior.
